# Supplementary figures and images for: The behaviour and activity budgets of two sympatric sloths; Bradypus variegatus and Choloepus hoffmanni
Source: PeerJ. 2023 May 29;11:e15430. doi: 10.7717/peerj.15430 (PMC10234273; doi:10.7717/peerj.15430)

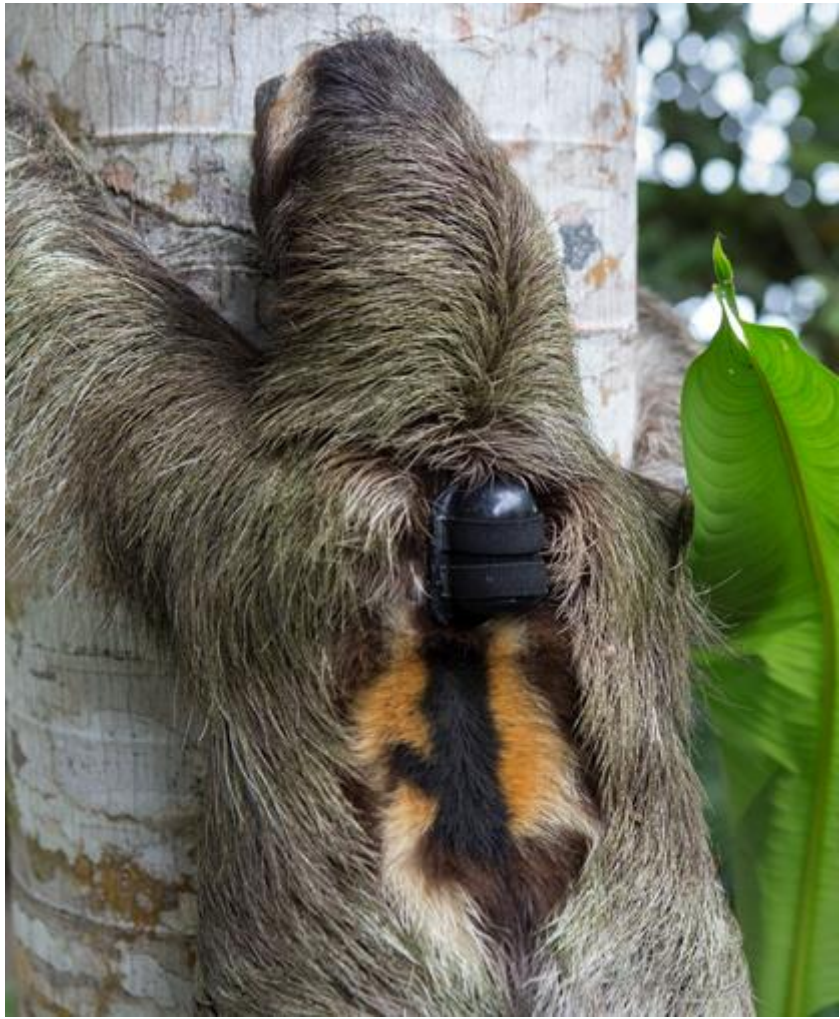

Supplement: Figure S1 — Backpack contains a Daily Diary data logger (DD) and Very High Frequency radio transmitter (VHF). Transmitter antenna is wrapped around the chest strap of the harness to avoid catching on branches. [file peerj-11-15430-s001.pdf]

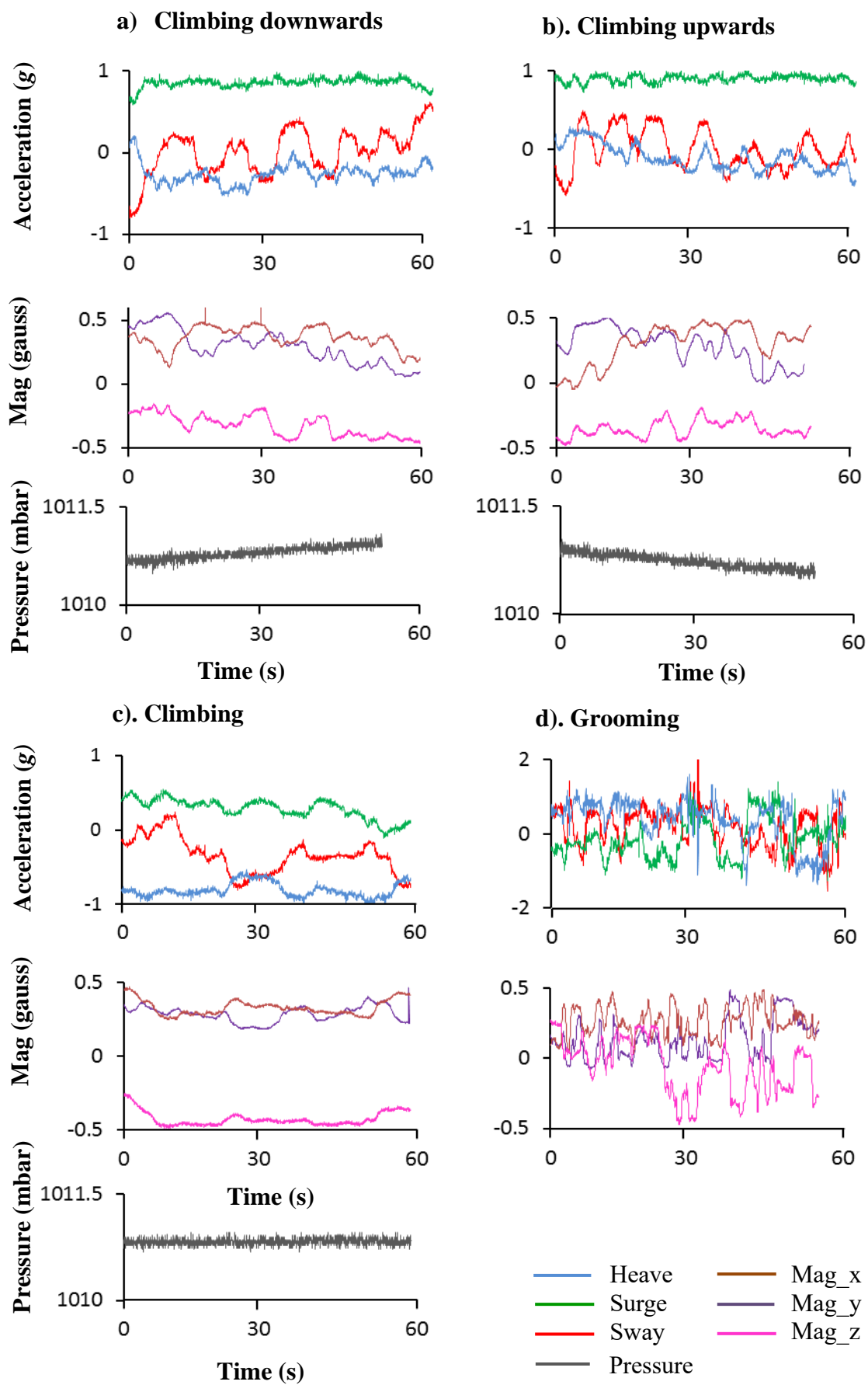

Supplement: Figure S2 — (a) Climbing downwards. Climbing activity is identified by characteristic wave forms in the sway signal, with mean values around 0, amplitudes of 0.5 g and periods between 5–20 s. Downwards climbing includes all climbing activity occurring for more than 15 s with a corresponding increase in pressure typically greater than 0.02 mbar per 5 s. (b) Climbing upwards. Climbing activity is identified by characteristic wave forms in the sway signal, with mean values around 0, amplitudes of 0.5 g and periods between 5–20 s. Upwards climbing includes all climbing activity occurring for more than 15 s with a corresponding decrease in pressure typically greater than −0.02 mbar per 5 s. (c) Climbing. Climbing (all horizontal climbing and vertical climbing for durations shorter than 15 s) identified by characteristic wave forms in the sway signal with mean values around 0 and periods between 5–20 s. In this case, surge acceleration always reads close to 1 and is relatively stable while the heave signal typically reads −1. Horizontal climbing is identified by stable pressure values. (d) Grooming. Behaviour identified by stability in pressure (not shown), apparently variable body position manifest in baseline levels of the acceleration and much more dynamism in the movement than shown for locomotion. Wave forms are not particularly obvious, but peak-to-peak periods are typically less than 5 s. [file peerj-11-15430-s002.pdf]

**a) Sleeping**

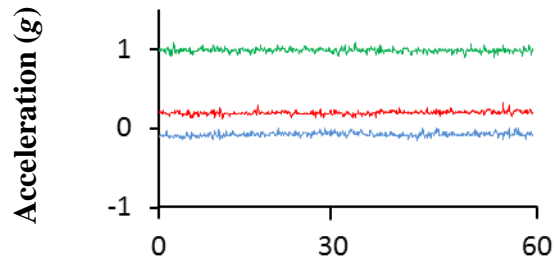

**b). Resting**

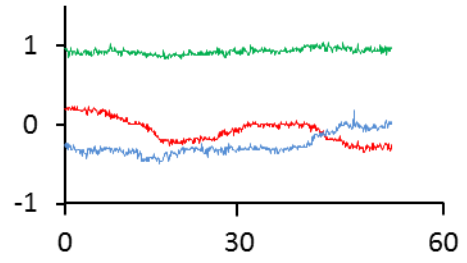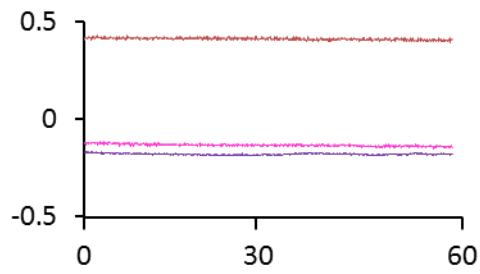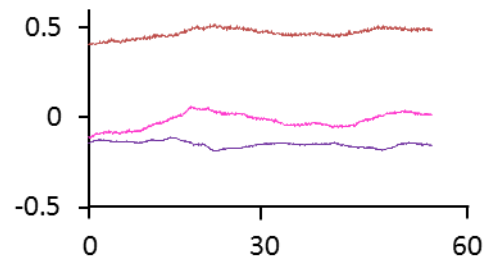

**Time (s)**

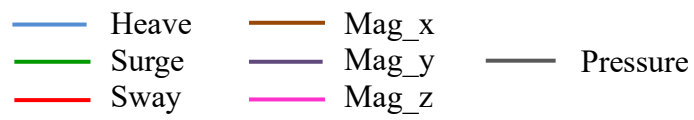

Supplement: Figure S3 — (a) Sleeping: Identified by static signals across all channels for duration longer than 10 min. (b) Resting. Static accelerometer and magnetometer signals lasting less than 10 min, or typically slow changes in surge and heave with wave forms (if visible) with amplitudes less than 0.5 g and periods of 30 s or greater. [file peerj-11-15430-s003.pdf]

Animal  
bv1:

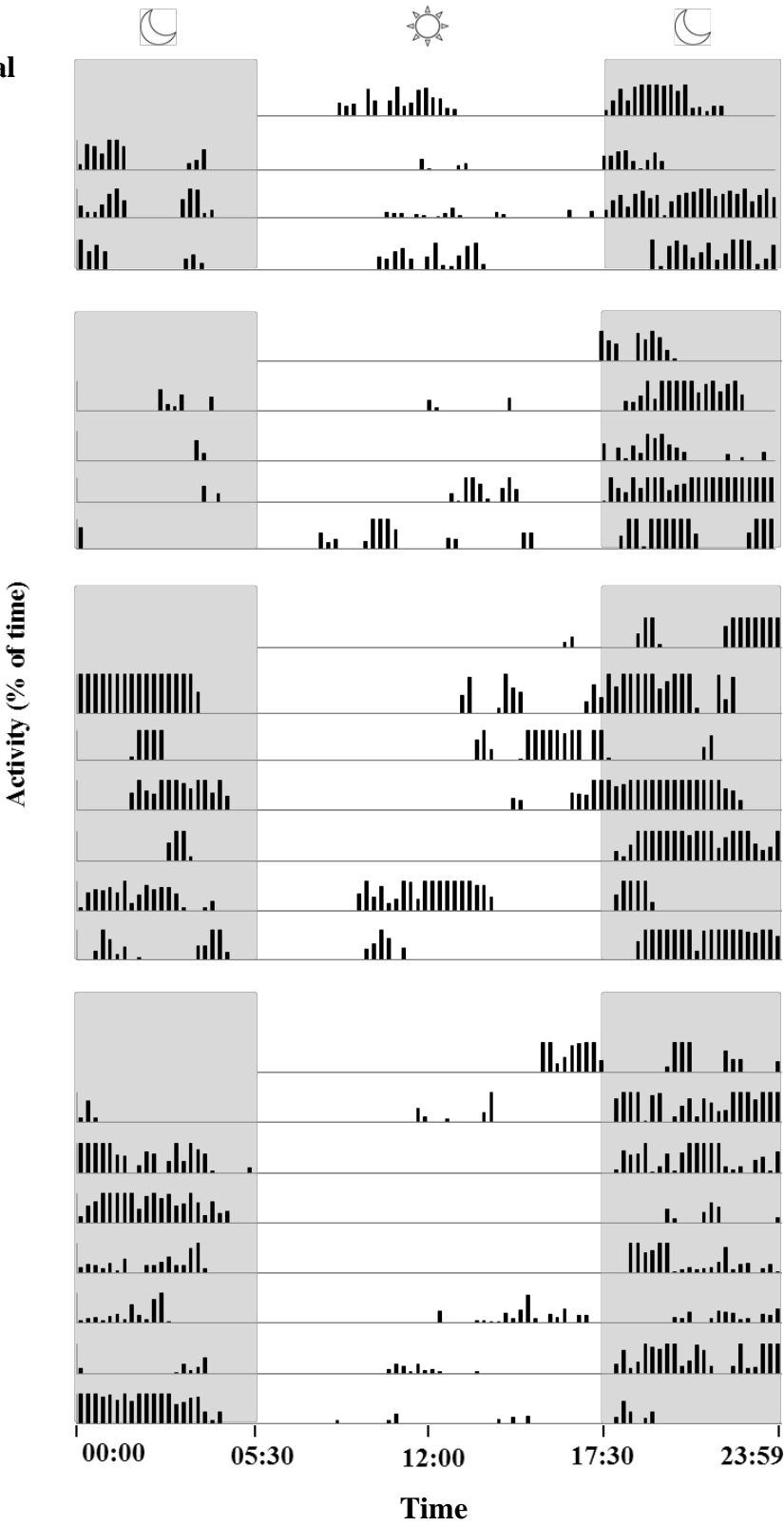

Supplement: Figure S4 — Each actogram represents a different dataset (#1 –4) and each row represents 24 h of data. Percent activity calculated every 15 min and dark phases shown by grey shading. [file peerj-11-15430-s004.pdf]

Animal  
bv2:

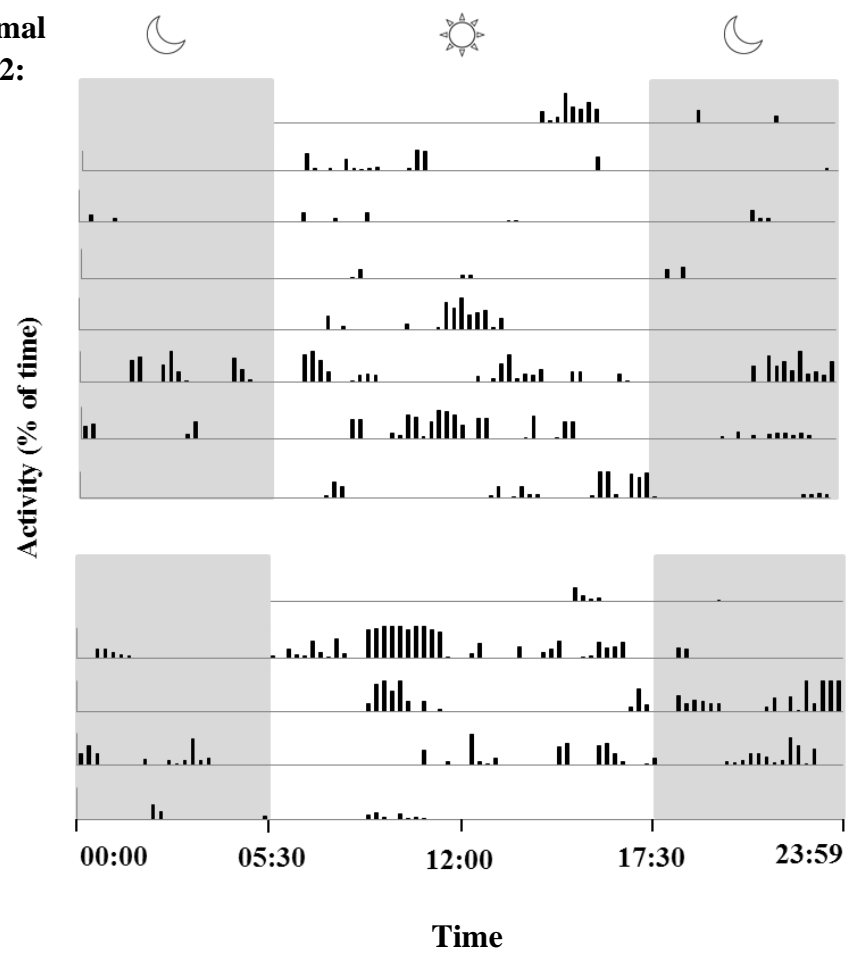

Supplement: Figure S5 — Each actogram represents a different dataset (#5 –6) and each row represents 24 h of data. Percent activity calculated every 15 min. Dark phases are shown by grey shading. [file peerj-11-15430-s005.pdf]

**Animal**  
**bv3:**

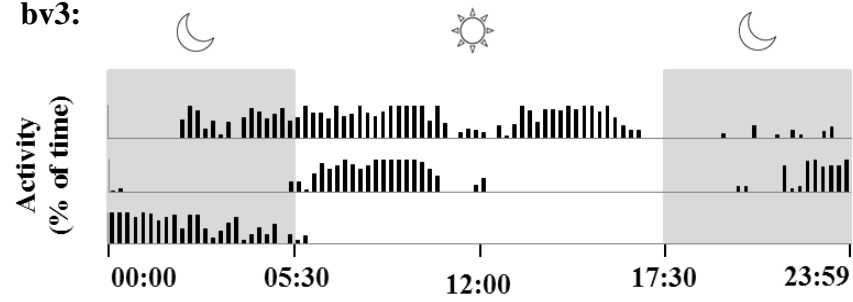

**Time**

Supplement: Figure S6 — Actogram represents dataset #7 and each row represents 24 h of data. Percent activity calculated every 15 min. Dark phases are shown by grey shading. [file peerj-11-15430-s006.pdf]

Animal  
bv4:

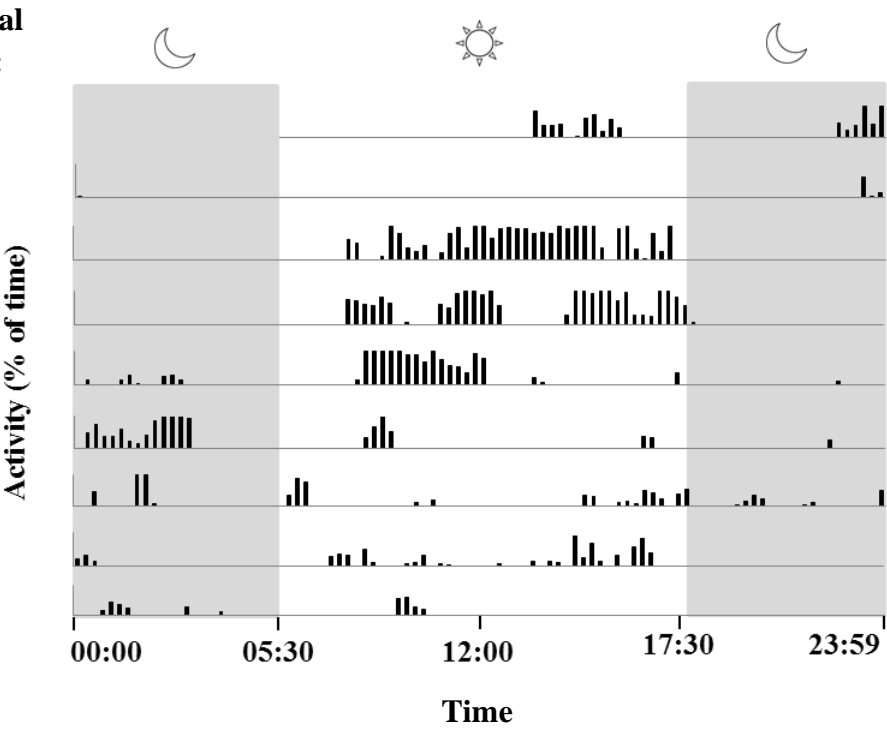

Supplement: Figure S7 — Actogram represents dataset #8 and each row represents 24 h of data. Percent activity calculated every 15 min. Dark phases are shown by grey shading. [file peerj-11-15430-s007.pdf]

Animal  
bv5:

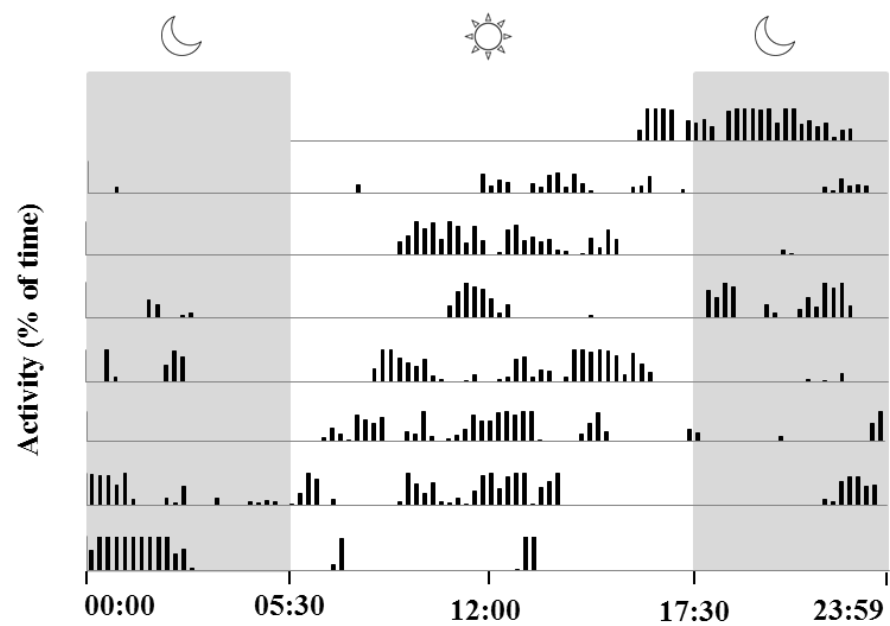

Supplement: Figure S8 — Actogram represents dataset #9 and each row represents 24 h of data. Percent activity calculated every 15 min and dark phases shown by grey shading. [file peerj-11-15430-s008.pdf]

**Animal**  
**bv6:**

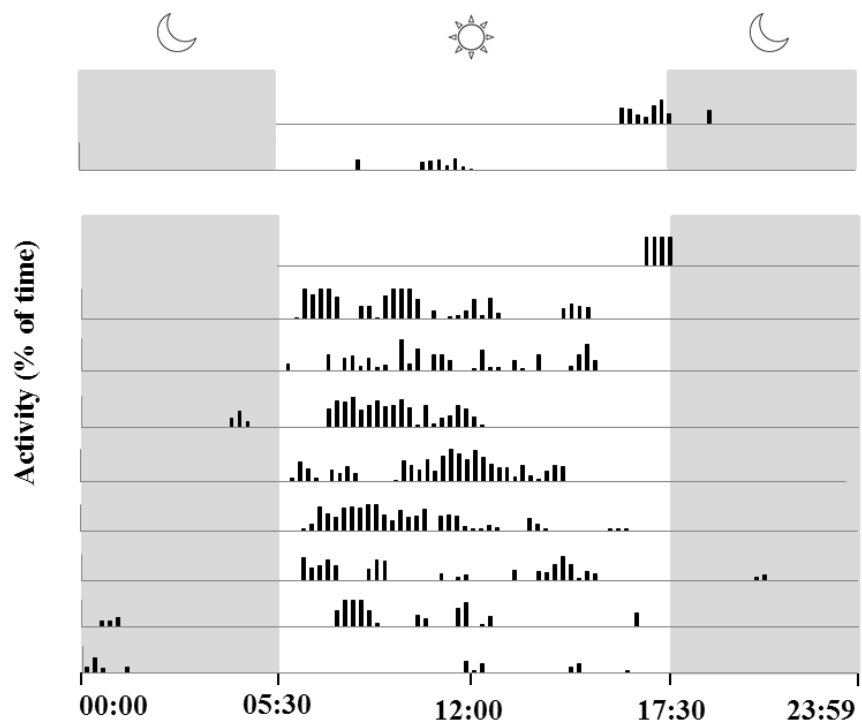

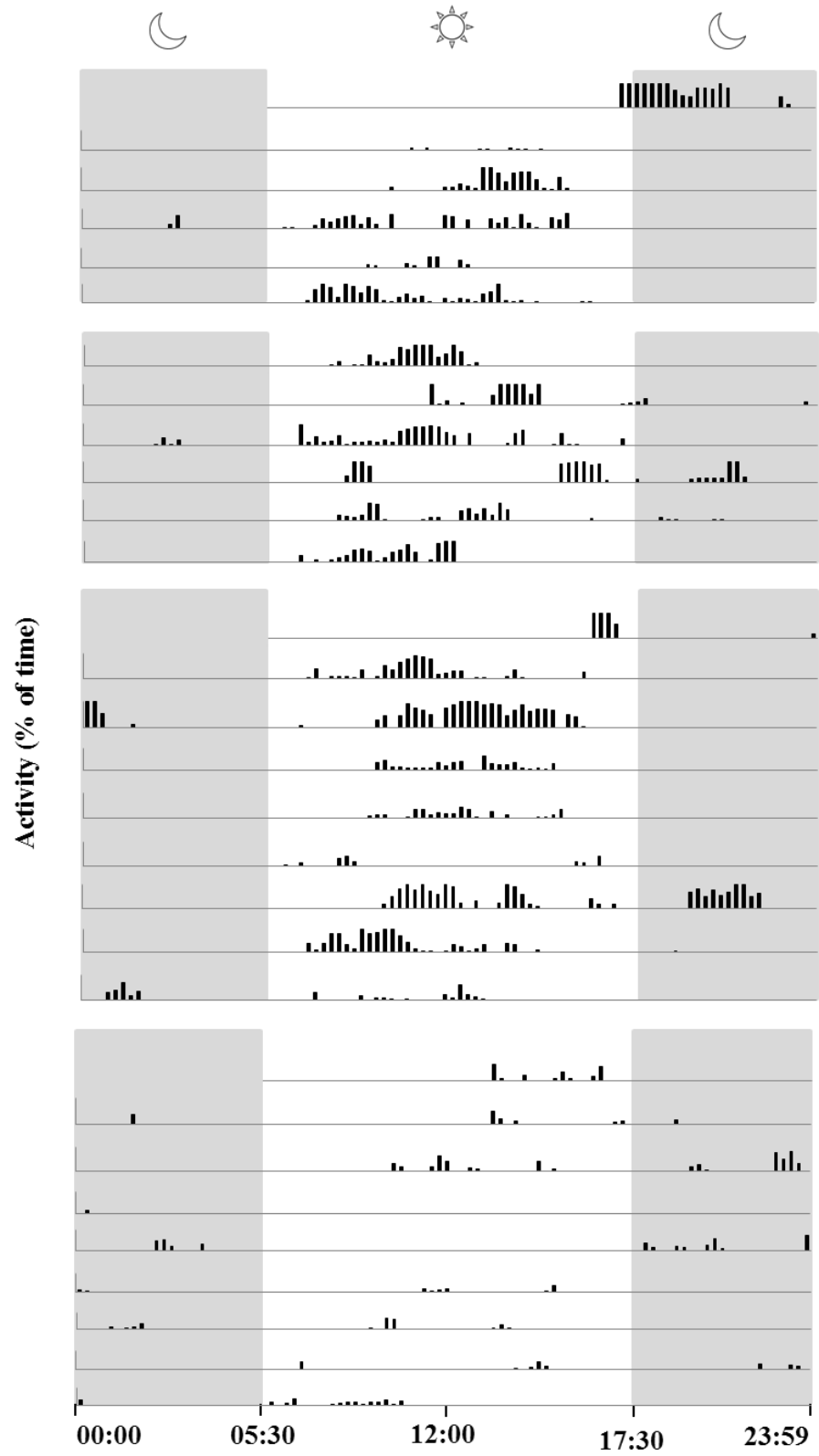

Supplement: Figure S9 — Each actogram represents a different dataset (#10 –15) and each row represents 24 h of data. Percent activity calculated every 15 min and dark phases shown by grey shading. [file peerj-11-15430-s009.pdf]

**Animal**  
**bv7:**

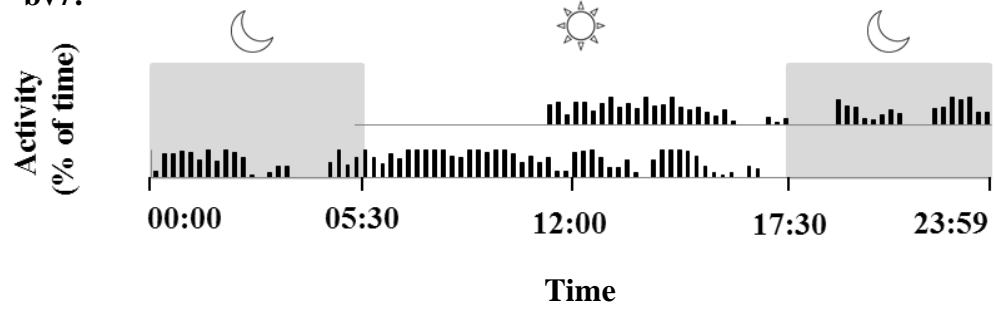

Supplement: Figure S10 — Actogram represents dataset #16 and each row represents 24 h of data. Percent activity calculated every 15 min. Dark phases are shown by grey shading. [file peerj-11-15430-s010.pdf]

**Animal**  
**bv8:**

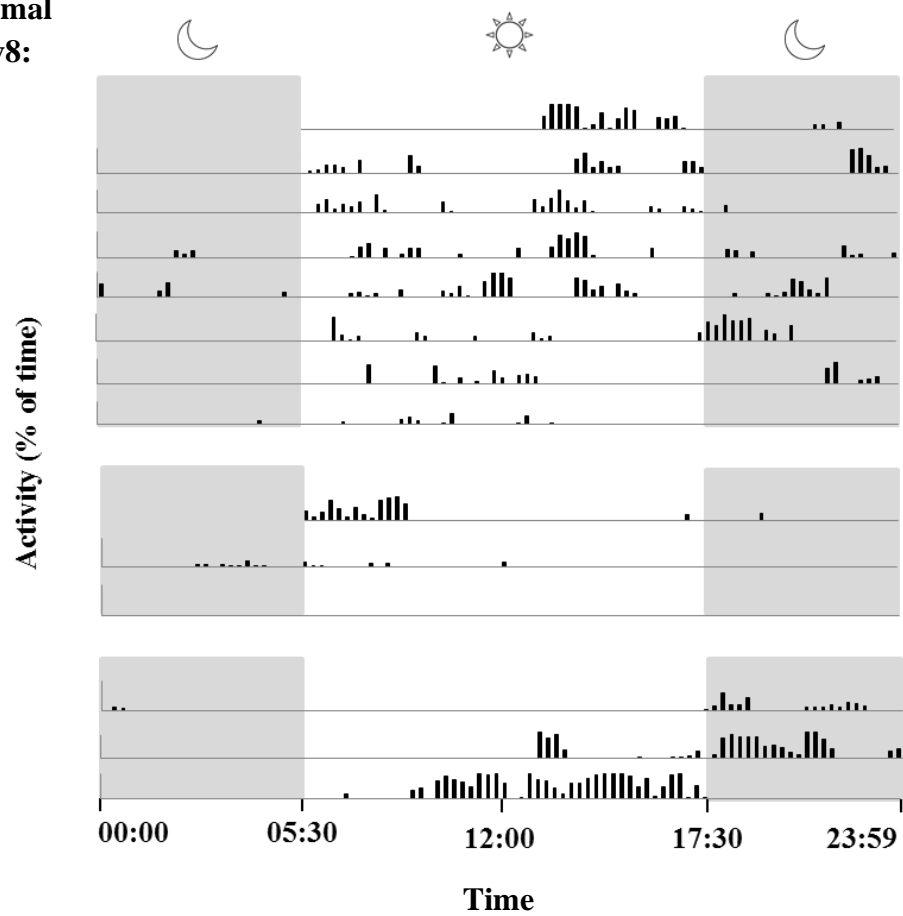

Supplement: Figure S11 — Each actogram represents a different dataset (#17 –19) and each row represents 24 h of data. Percent activity calculated every 15 min. Dark phases are shown by grey shading. [file peerj-11-15430-s011.pdf]

Animal  
ch1:

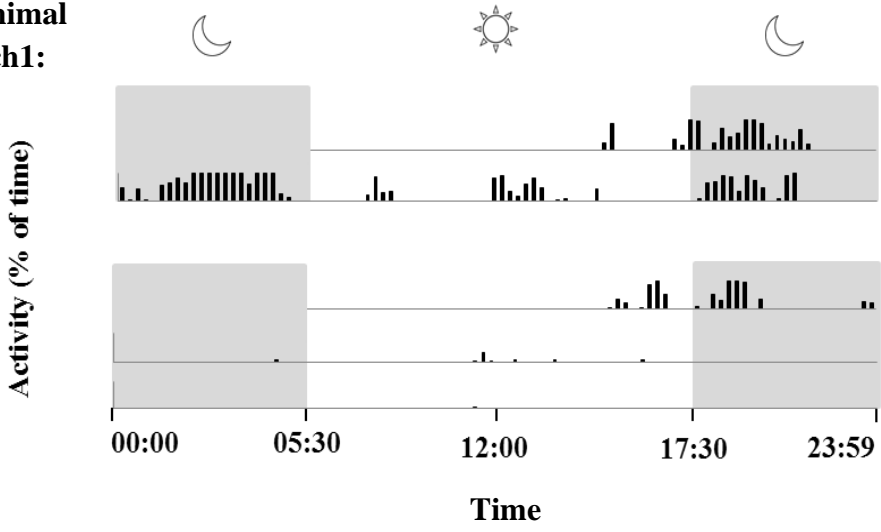

Supplement: Figure S12 — Each actogram represents a different dataset (#20 –21) and each row represents 24 h of data. Percent activity calculated every 15 min. Dark phases are shown by grey shading [file peerj-11-15430-s012.pdf]

**Animal  
ch2:**

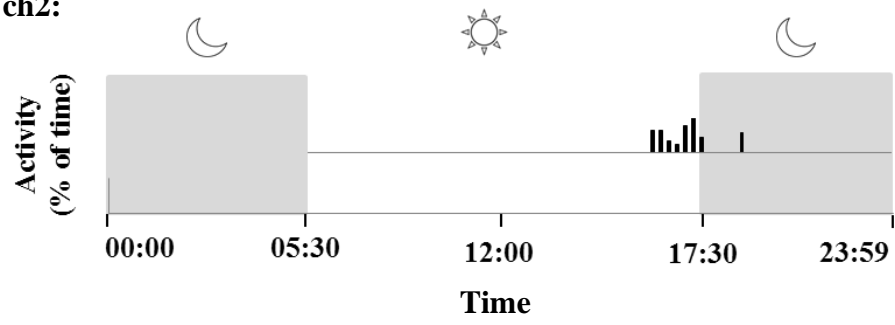

Supplement: Figure S13 — Actogram represents dataset #22 and each row represents 24 h, however only 12 h of data were collected for this individual. Percent activity calculated every 15 min. Dark phases are shown by grey shading [file peerj-11-15430-s013.pdf]

**Animal  
ch3:**

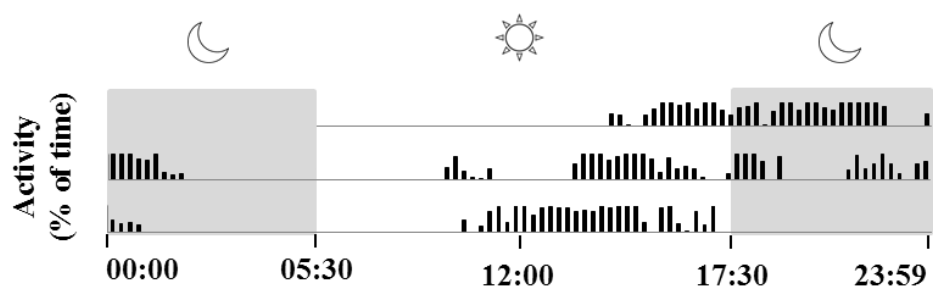

Supplement: Figure S14 — Actogram represents dataset #23 and each row represents 24 h of data. Percent activity calculated every 15 min and dark phases shown by grey shading. [file peerj-11-15430-s014.pdf]

**Animal  
ch4:**

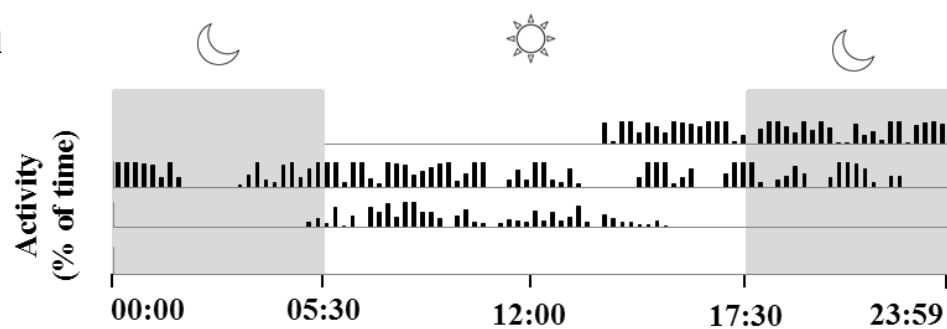

Supplement: Figure S15 — Actogram represents dataset #24 and each row represents 24 h of data. Percent activity calculated every 15 min and dark phases shown by grey shading. [file peerj-11-15430-s015.pdf]
