# Supplementary material for: The behaviour and activity budgets of two sympatric sloths; Bradypus variegatus and Choloepus hoffmanni
Source: PeerJ. 2023 May 29;11:e15430. doi: 10.7717/peerj.15430 (PMC10234273; doi:10.7717/peerj.15430)
Supplement: Table S1 [file peerj-11-15430-s016.docx]

| **Dataset #** | **Sloth** | **% time active** | **% time inactive** |
| --- | --- | --- | --- |
| 1 | bv1 | 20.9 | 79.1 |
| 2 |  | 18.8 | 81.2 |
| 3 |  | 29.0 | 71.0 |
| 4 |  | 19.0 | 81.0 |
| 5 | bv2 | 7.6 | 92.4 |
| 6 |  | 12.0 | 88.0 |
| 7 | bv3 | 38.1 | 61.9 |
| 8 | bv4 | 13.5 | 86.5 |
| 9 | bv5 | 20.4 | 79.6 |
| 10 | bv6 | 10.1 | 89.9 |
| 11 |  | 11.5 | 88.5 |
| 12 |  | 10.4 | 89.6 |
| 13 |  | 2.9 | 97.1 |
| 14 |  | 5.7 | 94.3 |
| 15 |  | 11.2 | 88.8 |
| 16 | bv7 | 50.7 | 49.3 |
| 17 | bv8 | 9.7 | 90.3 |
| 18 |  | 6.2 | 93.8 |
| 19 |  | 17.4 | 82.6 |
| 20 | ch1 | 32.8 | 67.2 |
| 21 |  | 4.8 | 95.2 |
| 22 | ch2 | 62.2 | 37.8 |
| 23 | ch3 | 33.2 | 66.8 |
| 24 | ch4 | 30.1 | 69.9 |
