# Supplementary material for: The behaviour and activity budgets of two sympatric sloths; Bradypus variegatus and Choloepus hoffmanni
Source: PeerJ. 2023 May 29;11:e15430. doi: 10.7717/peerj.15430 (PMC10234273; doi:10.7717/peerj.15430)
Supplement: Table S2 [file peerj-11-15430-s017.docx]

|  | % of total time in behaviour | | | | | |
| --- | --- | --- | --- | --- | --- | --- |
|  | sleeping | resting | grooming | climbing | climbing up | climbing down |
|  |  |  |  |  |  |  |
| Bv1 | 52.0 | 25.7 | 0.1 | 18.9 | 2.1 | 1.3 |
| Bv2 | 75.3 | 15.7 | 0.3 | 7.5 | 0.6 | 0.7 |
| Bv3 | 29.0 | 31.3 | 0.0 | 33.5 | 4.0 | 2.3 |
| Bv4 | 67.6 | 19.1 | 0.1 | 10.8 | 1.5 | 1.1 |
| Bv5 | 53.0 | 26.8 | 0.1 | 15.3 | 3.0 | 1.7 |
| Bv6 | 68.8 | 22.4 | 0.1 | 7.4 | 0.7 | 0.5 |
| Bv7 | 14.9 | 33.8 | 0.0 | 37.5 | 8.3 | 5.5 |
| Bv8 | 67.0 | 22.6 | 0.1 | 8.1 | 1.5 | 0.7 |
| Ch1 | 52.06 | 14.47 | 0 | 27.05 | 4.59 | 1.83 |
| Ch2 | 49.54 | 20.34 | 0.39 | 26.18 | 2.3 | 1.25 |
| Ch3 | 71.7 | 12.21 | 0.32 | 13.35 | 1.93 | 0.5 |
| Ch4 | 16.73 | 21.7 | 0.04 | 47.41 | 11.15 | 2.96 |
