# Supplementary material for: The behaviour and activity budgets of two sympatric sloths; Bradypus variegatus and Choloepus hoffmanni
Source: PeerJ. 2023 May 29;11:e15430. doi: 10.7717/peerj.15430 (PMC10234273; doi:10.7717/peerj.15430)
Supplement: Table S3 [file peerj-11-15430-s018.docx]

| Sloth | Sleeping | SD | Resting | SD | Grooming | SD | Climbing | SD | Climbing upwards | SD | Climbing downwards | SD | Inactivity | SD | Activity | SD |
| --- | --- | --- | --- | --- | --- | --- | --- | --- | --- | --- | --- | --- | --- | --- | --- | --- |
| bv1 | 113.09 | 112.13 | 14.10 | 39.55 | 1.03 | 1.13 | 9.25 | 15.34 | 6.51 | 9.16 | 6.20 | 7.98 | 34.07 | 73.14 | 8.40 | 14.09 |
| bv2 | 106.99 | 101.53 | 9.49 | 12.12 | 1.59 | 1.98 | 4.56 | 6.21 | 3.34 | 2.27 | 3.09 | 2.61 | 38.59 | 71.80 | 4.08 | 5.54 |
| bv3 | 119.51 | 109.02 | 7.32 | 11.53 | 0.37 | 0.30 | 7.59 | 7.33 | 7.24 | 5.25 | 6.23 | 4.15 | 13.35 | 36.49 | 7.34 | 6.96 |
| bv4 | 93.16 | 85.80 | 9.17 | 14.13 | 0.74 | 0.67 | 4.85 | 5.00 | 2.74 | 1.79 | 2.65 | 1.88 | 30.91 | 58.25 | 4.11 | 4.39 |
| bv5 | 62.54 | 68.65 | 8.17 | 12.12 | 0.91 | 0.58 | 5.20 | 6.78 | 2.88 | 2.68 | 2.41 | 1.84 | 19.32 | 39.46 | 4.17 | 5.64 |
| bv6 | 148.30 | 132.54 | 11.13 | 20.31 | 0.52 | 0.38 | 4.29 | 6.76 | 5.84 | 4.19 | 5.72 | 4.26 | 36.79 | 80.46 | 4.13 | 6.37 |
| bv7 | 73.34 | 55.18 | 6.65 | 7.41 | 0.29 | 0.18 | 7.03 | 7.61 | 10.26 | 7.38 | 7.19 | 7.06 | 9.22 | 17.54 | 7.28 | 7.54 |
| bv8 | 120.63 | 103.74 | 9.99 | 15.31 | 0.76 | 1.00 | 4.17 | 4.65 | 9.95 | 6.75 | 5.95 | 4.08 | 31.79 | 65.05 | 4.39 | 4.94 |
| ch1 | 109.76 | 118.00 | 6.67 | 10.36 | 0.96 | 1.47 | 7.05 | 8.41 | 5.91 | 4.75 | 5.70 | 3.73 | 33.80 | 75.70 | 6.11 | 7.61 |
| ch2 | 119.93 | NA | 4.20 | 4.27 | 0.32 | NA | 6.18 | 5.06 | 6.66 | 4.00 | 5.30 | 2.16 | 7.25 | 19.24 | 6.13 | 4.77 |
| ch3 | 149.84 | 142.31 | 5.35 | 5.58 | NA | NA | 8.06 | 9.99 | 7.93 | 7.64 | 4.31 | 1.78 | 21.81 | 65.11 | 7.67 | 9.24 |
| ch4 | 169.78 | 158.02 | 6.25 | 7.21 | 0.69 | 0.46 | 7.67 | 9.33 | 7.33 | 3.79 | 4.65 | 4.05 | 19.70 | 63.15 | 6.61 | 8.51 |
| Bradypus (mean) | 116.35 | 112.81 | 10.63 | 22.84 | 0.80 | 1.04 | 5.82 | 9.37 | 5.15 | 6.09 | 4.43 | 5.00 | 31.86 | 69.02 | 5.37 | 8.60 |
| Choloepus (mean) | 131.48 | 131.69 | 5.94 | 7.61 | 0.78 | 0.95 | 7.40 | 8.79 | 6.97 | 5.27 | 4.75 | 3.03 | 23.05 | 65.07 | 6.66 | 8.05 |
| Overall (mean) | 117.34 | 114.11 | 10.16 | 21.85 | 0.80 | 1.02 | 6.00 | 9.32 | 5.36 | 6.02 | 4.46 | 4.85 | 31.04 | 68.70 | 5.52 | 8.55 |
