# Supplementary material for: The behaviour and activity budgets of two sympatric sloths; Bradypus variegatus and Choloepus hoffmanni
Source: PeerJ. 2023 May 29;11:e15430. doi: 10.7717/peerj.15430 (PMC10234273; doi:10.7717/peerj.15430)
Supplement: Table S4 — Periods of the day separated into: dawn (04:00–07:00), day (07:00–16:00), dusk (16:00–19:00) and night (19:00–04:00). Based on the significance level, sloths are identified as either nocturnal (significantly higher activity during the night compared to the day), diurnal (significantly higher activity during the day compared to the night) or cathemeral (no significant difference in activity [Kruskal Wallis test]). [file peerj-11-15430-s019.docx]

| **Sloth** | **N** | **Kruskal Wallis test** | | | **Dunn's test post hoc** | | | **Activity preference** |
| --- | --- | --- | --- | --- | --- | --- | --- | --- |
|  |  | **X²** | **df** | **p** | **Time of day** | **Z value** | **p value** |  |
| bv1 | 525 | 91.413 | 3 | <0.001* | dawn-day | -0.38346 | 0.3507 | Nocturnal |
|  |  |  |  |  | dawn -dusk | -2.37512 | 0.0088 |  |
|  |  |  |  |  | day-dusk | -2.55467 | 0.0053 |  |
|  |  |  |  |  | dawn-night | -6.53962 | 0.0000* |  |
|  |  |  |  |  | day-night | -8.781 | 0.0000* |  |
|  |  |  |  |  | dusk-night | -3.86187 | 0.0001* |  |
| bv2 | 275 | 20.385 | 3 | <0.001* | dawn-day | -4.19965 | 0.0000* | Diurnal |
|  |  |  |  |  | dawn -dusk | -2.54674 | 0.0054 |  |
|  |  |  |  |  | day-dusk | 1.179952 | 0.1190 |  |
|  |  |  |  |  | dawn-night | -2.13037 | 0.0166 |  |
|  |  |  |  |  | day-night | 3.001548 | 0.0013* |  |
|  |  |  |  |  | dusk-night | 0.980239 | 0.1635 |  |
| bv3 | 55 | 9.1171 | 3 | 0.028 | dawn-day | -0.68947 | 0.2453 | Cathemeral |
|  |  |  |  |  | dawn -dusk | 1.943853 | 0.0260 |  |
|  |  |  |  |  | day-dusk | 2.770393 | 0.0028* |  |
|  |  |  |  |  | dawn-night | 0.914209 | 0.1803 |  |
|  |  |  |  |  | day-night | 2.023823 | 0.0215 |  |
|  |  |  |  |  | dusk-night | -1.43902 | 0.0751 |  |
| bv4 | 216 | 21.283 | 3 | <0.001* | dawn-day | -4.00048 | 0.0000* | Diurnal |
|  |  |  |  |  | dawn -dusk | -3.26261 | 0.0006* |  |
|  |  |  |  |  | day-dusk | 0.004617 | 0.4982 |  |
|  |  |  |  |  | dawn-night | -1.89993 | 0.0287 |  |
|  |  |  |  |  | day-night | 2.970621 | 0.0015* |  |
|  |  |  |  |  | dusk-night | 2.095928 | 0.0180 |  |
| bv5 | 166 | 22.278 | 3 | <0.001* | dawn-day | -4.47064 | 0.0000* | Cathemeral |
|  |  |  |  |  | dawn -dusk | -1.6076 | 0.0540 |  |
|  |  |  |  |  | day-dusk | 2.509761 | 0.0060 |  |
|  |  |  |  |  | dawn-night | -2.7133 | 0.0033* |  |
|  |  |  |  |  | day-night | 2.490773 | 0.0064 |  |
|  |  |  |  |  | dusk-night | -0.74441 | 0.2283 |  |
| bv6 | 967 | 191.93 | 3 | <0.001* | dawn-day | -9.80849 | 0.0000* | Diurnal |
|  |  |  |  |  | dawn -dusk | -2.91408 | 0.0018* |  |
|  |  |  |  |  | day-dusk | 6.292946 | 0.0000* |  |
|  |  |  |  |  | dawn-night | -0.9551 | 0.1698 |  |
|  |  |  |  |  | day-night | 12.56601 | 0.0000* |  |
|  |  |  |  |  | dusk-night | 2.621574 | 0.0044* |  |
| bv7 | 48 | 9.1864 | 3 | 0.02691 | dawn-day | -2.07563 | 0.0190 | Cathemeral |
|  |  |  |  |  | dawn -dusk | -0.07414 | 0.4704 |  |
|  |  |  |  |  | day-dusk | 1.984816 | 0.0236 |  |
|  |  |  |  |  | dawn-night | -0.24648 | 0.4027 |  |
|  |  |  |  |  | day-night | 2.586801 | 0.0048* |  |
|  |  |  |  |  | dusk-night | -0.15567 | 0.4381 |  |
| bv8 | 336 | 15.7863 | 3 | 0.0013* | dawn-day | -2.86974 | 0.0021* | Diurnal |
|  |  |  |  |  | dawn -dusk | -2.10676 | 0.0176 |  |
|  |  |  |  |  | day-dusk | 0.289495 | 0.3861 |  |
|  |  |  |  |  | dawn-night | -0.52261 | 0.3006 |  |
|  |  |  |  |  | day-night | 3.319349 | 0.0005* |  |
|  |  |  |  |  | dusk-night | 2.057639 | 0.0198 |  |
| ch1 | 120 | 2.9531 | 3 | 0.3989 | dawn-day | -0.45468 | 0.3247 | Cathemeral |
|  |  |  |  |  | dawn -dusk | -1.54758 | 0.0609 |  |
|  |  |  |  |  | day-dusk | -1.44071 | 0.0748 |  |
|  |  |  |  |  | dawn-night | -0.91201 | 0.1809 |  |
|  |  |  |  |  | day-night | -0.64676 | 0.2589 |  |
|  |  |  |  |  | dusk-night | 0.983383 | 0.1627 |  |
| ch2 | 14 | 9.2146 | 2 | 0.0099 | dawn -dusk | -2.35069 | 0.0094 | NA (insufficient data) |
|  |  |  |  |  | dawn-night | -0.27663 | 0.3910 |  |
|  |  |  |  |  | dusk-night | 2.894439 | 0.0019 |  |
| ch3 | 52 | 12.8911 | 3 | 0.0048* | dawn-day | -2.55404 | 0.0053 | Cathemeral |
|  |  |  |  |  | dawn -dusk | -3.57556 | 0.0002* |  |
|  |  |  |  |  | day-dusk | -1.77416 | 0.0380 |  |
|  |  |  |  |  | dawn-night | -2.58048 | 0.0049* |  |
|  |  |  |  |  | day-night | -0.08499 | 0.4661 |  |
|  |  |  |  |  | dusk-night | 1.681669 | 0.0463 |  |
| ch4 | 76 | 1.2671 | 3 | 0.737 | dawn-day | -0.33309 | 0.3695 | Cathemeral |
|  |  |  |  |  | dawn -dusk | -0.06879 | 0.4726 |  |
|  |  |  |  |  | day-dusk | 0.271603 | 0.3930 |  |
|  |  |  |  |  | dawn-night | 0.44523 | 0.3281 |  |
|  |  |  |  |  | day-night | 1.116039 | 0.1322 |  |
|  |  |  |  |  | dusk-night | 0.56553 | 0.2859 |  |
